# Molecular drivers of tumor progression in microsatellite stable *APC* mutation-negative colorectal cancers

**DOI:** 10.1038/s41598-021-02806-x

**Published:** 2021-12-06

**Authors:** Adam Grant, Rosa M. Xicola, Vivian Nguyen, James Lim, Curtis Thorne, Bodour Salhia, Xavier Llor, Nathan Ellis, Megha Padi

**Affiliations:** 1 University of Arizona Cancer Center, University of Arizona, 1515 N. Campbell Avenue, Tucson, AZ 85724 USA; 2 Department of Medicine and Cancer Center, Yale University, New Haven, CT USA; 3 Department of Molecular and Cellular Biology, University of Arizona, Tucson, AZ USA; 4 Department of Cellular and Molecular Medicine, University of Arizona, Tucson, AZ USA; 5 Department of Translational Genomics, University of Southern California, Los Angeles, CA USA

**Keywords:** Colorectal cancer, Cancer genomics

## Abstract

The tumor suppressor gene adenomatous polyposis coli (*APC*) is the initiating mutation in approximately 80% of all colorectal cancers (CRC), underscoring the importance of aberrant regulation of intracellular WNT signaling in CRC development. Recent studies have found that early-onset CRC exhibits an increased proportion of tumors lacking an *APC* mutation. We set out to identify mechanisms underlying *APC* mutation-negative (*APC*^*mut–*^) CRCs. We analyzed data from The Cancer Genome Atlas to compare clinical phenotypes, somatic mutations, copy number variations, gene fusions, RNA expression, and DNA methylation profiles between *APC*^*mut–*^ and *APC* mutation-positive (*APC*^*mut*+^) microsatellite stable CRCs. Transcriptionally, *APC*^*mut–*^ CRCs clustered into two approximately equal groups. Cluster One was associated with enhanced mitochondrial activation. Cluster Two was strikingly associated with genetic inactivation or decreased RNA expression of the WNT antagonist *RNF43*, increased expression of the WNT agonist *RSPO3*, activating mutation of *BRAF*, or increased methylation and decreased expression of *AXIN2*. *APC*^*mut–*^ CRCs exhibited evidence of increased immune cell infiltration, with significant correlation between M2 macrophages and *RSPO3*. *APC*^*mut–*^ CRCs comprise two groups of tumors characterized by enhanced mitochondrial activation or increased sensitivity to extracellular WNT, suggesting that they could be respectively susceptible to inhibition of these pathways.

## Introduction

Colorectal cancer (CRC) is the second deadliest cancer in the United States, with an estimated 147,950 individuals diagnosed and 53,200 deaths in 2020^1^. Although there have been great reductions in CRC incidence and mortality widely attributed to increased screening^2^, the incidence of CRC has been increasing in individuals less than 50 years of age at a rate of 2% per year since 1994^3^. Molecular analysis has shown that < 20% of early-onset CRC cases are explained by genetically determined hereditary syndromes^4^ and a variety of environmental factors have been postulated to underlie its increase^5^, suggesting that a unitary cause of early-onset CRC will be elusive. With early-onset CRC manifesting as a heterogenous disease caused by a multitude of factors, there is a pressing need to identify the distinct molecular subtypes of CRC that are overrepresented in early-onset cases.

Somatic mutation of the adenomatous polyposis coli (*APC*) gene is the initiating event in approximately 80% of all CRCs, but *APC* mutations are significantly less frequent in early-onset CRCs^6–8^. *APC* is a structural and regulatory component of a destruction complex, which responds to WNT stimulation by inhibition of degradation of the stem cell-promoting transcription factor β-catenin, encoded by the *CTNNB1* gene^9^. Failure to regulate β-catenin by degradation due to mutational inactivation of *APC* throws colorectal epithelial cells into a continuous “WNT-activated” state; they no longer require activation by WNTs to maintain the stem cell compartment^10^. The fact that early-onset CRCs more frequently lack an *APC* mutation suggests that many of these tumors depend on alternative molecular mechanisms. In mismatch repair-deficient CRCs, which exhibit microsatellite instability (MSI) and constitute 12–15% of all CRCs, *APC* mutations are also significantly less frequent and *BRAF* mutations constitute a dominant driver mechanism^11^. What initiates and drives the carcinogenetic process in microsatellite stable (MSS) CRCs that lack *APC* mutations? Here, we comprehensively compare molecular profiles of MSS *APC* mutation-positive CRCs (*APC*^*mut*+^) and MSS *APC* mutation-negative (*APC*^*mut–*^) CRCs to identify novel *APC*-independent mechanisms driving CRC subtypes.

## Methods

### Analyses of genomic alterations in case series

To formulate a discovery series, we obtained colon adenocarcinoma (COAD) and rectal adenocarcinoma (READ) data from The Cancer Genome Atlas (TCGA) in the Genomic Data Commons portal (Supplementary File 1). Curated somatic nucleotide variant and copy number data were extracted using TCGAbiolinks and FireBrowse, respectively^12^. Deep deletions, amplifications, and gene fusions^13^ were identified. We excluded hypermutable cases by removing MSI-high cases based on clinical data and by removing cases with > 700 mutations. CRC samples were classified as *APC*^*mut–*^ if they lacked a non-silent mutation or deep (homozygous) deletion in *APC* or lacked a mutation in *CTNNB1*^14^. With these filtration steps, we had 63 *APC*^*mut–*^ samples and 362 *APC*^*mut*+^ samples. We compared genomic alterations between *APC*^*mut–*^ and *APC*^*mut*+^ CRCs by Fisher’s exact test and tested mutual exclusivity by CoMEt^15^. For more details on the bioinformatics analysis, see Supplementary Methods.

For validation series, we used the CPTAC-2^16^ and GSE35896^17^ datasets, because they were the only CRC datasets with *APC* mutation status and gene expression data available from the cBioPortal, the International Cancer Genome Consortium or studies utilized by Guinney et al*.* to determine consensus molecular subtypes of CRC^18^. CPTAC-2 was downloaded from cBioPortal and GSE3896 from synapse.org^18^. In the CPTAC-2 dataset, we identified 11 *APC*^*mut–*^ CRCs and 70 *APC*^*mut*+^ CRCs. Because GSE35896 did not have whole exome sequencing or copy number data, we could not filter for hypermutation, *APC* deep deletion, or *CTNNB1* mutations. Based on the data available, we classified 16 out of 56 MSS CRC samples as *APC*^*mut–*^.

### Transcriptomic analyses

For TCGA and CPTAC-2 series, we obtained HTSeq count files and used the edgeR and limma pipeline to normalize the counts matrix. For GSE35896, we used the RMA normalized data. Genes that had less than one count per million in more than half the samples were discarded. MBatch analysis showed no evidence of batch effects.

We used limma to identify differentially expressed genes between *APC*^*mut–*^ and *APC*^*mut*+^ CRCs (P_adj_ < 0.05). PathView was used to map differentially expressed genes onto the KEGG WNT canonical signaling pathway, with the node sum parameter set to “max.abs”^19^. Gene set enrichment analysis (GSEA) was performed using fgsea^20^. For GSEA input, we used the Gene Ontology (GO) biological process gene sets from MSigDB and ranked the genes by the t-statistic from our differential expression analysis. The Cytoscape application EnrichmentMap was used to visualize all statistically significant GO terms (P_adj_ < 0.05)^21^. CIBERSORTx was used to impute the fraction of immune cells based on gene expression data from the TCGA, GSE35896, and CPTAC-2 datasets^22^.

To characterize the theoretical WNT ligand sensitivity of *APC*^*mut–*^ tumors, we defined a score by the normalized expression of *RSPO3* minus the sum of the normalized expression levels of *RNF43* and *ZNRF3*:

$$ {\text{WNT}}_{{{\text{LS}}}} = {\text{Z}}\left[ {RSPO3_{mRNA - z} - (RNF43_{mRNA - z} + ZNRF3_{mRNA - z} )} \right], $$

where the subscript mRNA-z indicates the z-score of the expression value relative to all samples including tumors and normals*.* The WNT_LS_ score for each tumor was then compared to the maximum WNT ligand expression over all 12 WNTs in the same tumor.

### DNA methylation analyses

Methylation was assayed by TCGA using Illumina Human Methylation 450 arrays and data was accessed using TCGAbiolinks. Preprocessing and normalization were carried out with the R package minfi^23^. MBatch analysis showed no evidence of batch effects. Differentially methylated regions (DMRs) were identified using DMRcate and annotated with annotatr^24,25^. For DMRs that spanned multiple gene regions, we selected the gene with the most significant beta-values. To quantify methylation of a DMR, we took the average of all the statistically significant beta-values associated with the DMR.

### DepMap data analyses

DepMap data was obtained from https://depmap.org/portal/download/^26^. CRC cell lines were selected excluding those with MSI and with > 800 mutations. To distinguish *APC*^*mut–*^ from *APC*^*mut*+^ CRC cell lines, we used the same filtering steps we used for the TCGA dataset. To assess the effect of CRISPR knockouts, we applied a Welch’s two-sample t-test statistic to the dependency scores of *APC*^*mut–*^ and *APC*^*mut*+^ cell lines. Dependency scores were extracted from the file “Achilles_gene_dependency.csv” on the DepMap portal.

### Ethics statement

Ethics approval is not required for this study because it does not involve human participants or animal subjects.

## Results

### Age effect in ***APC***^***mut–***^ CRCs

To identify characteristics that distinguished *APC*^*mut–*^ from *APC*^*mut*+^ CRCs, we compared molecular profiles between the two groups in a discovery series from the TCGA, then validated the results in two additional publicly available series. CRC samples that exhibited MSI or were hypermutated were excluded from our study, because tumors with these characteristics constitute a well-defined subtype^11^. In addition to separating MSS and non-hypermutated CRCs by *APC* mutation status, samples that contained a *CTNNB1* mutation^14^ or deep deletion of *APC* were also classified as *APC*^*mut*+^. After applying these filtration steps, we classified 63 of 425 (15%) of the MSS CRCs in the TCGA dataset as *APC*^*mut–*^. In the GSE35896 validation dataset, 16 out of 56 (29%) CRCs were classified as *APC*^*mut–*^ and in the CPTAC-2 dataset, 11 out of 81 (14%) CRCs were classified as *APC*^*mut–*^.

We tested clinical features that might be statistically associated with TCGA *APC*^*mut–*^ CRCs (Table 1). In agreement with previous studies^6–8^, *APC*^*mut–*^ CRCs were diagnosed at a younger age (61.4 in *APC*^*mut–*^ vs. 66.4 in *APC*^*mut*+^), and 63% of tumors diagnosed < 50 were *APC*^*mut–*^. *APC*^*mut–*^ CRCs were also younger in the CPTAC-2 dataset (61.5 in *APC*^*mut–*^ vs. 65.5 in *APC*^*mut*+^), but this observation did not reach statistical significance (p = 0.24). (Age of diagnosis was not available for the GSE35896 dataset.) In addition to age, TCGA *APC*^*mut–*^ CRCs were more prevalent in Asians (p = 0.005), were more likely to be classified as CpG island methylator phenotype (CIMP) high (p = 0.02), and were more likely to be diagnosed later than stage one (p = 0.035).

**Table 1:** Comparison of clinical features in *APC* mutation-positive (*APC*^*mut*+^) and *APC* mutation-negative (*APC*^*mut–*^) colorectal cancers.

Feature	*APC*^*mut*+^ (N = 362, 85%)	*APC*^*mut–*^ (N = 63, 15%)	*P* value
Age	66.4	61.4	**.004**
Non-silent mutations	121.4	112.4	.21
Male/female	194/165 (54%)	30/33 (48%)	.41
COAD/READ	250/109 (70%)	51/12 (81%)	.07
Proximal/distal	104/195 (35%)	24/25 (49%)	.08
White	305/359 (85%)	53/63 (84%)	.85
African American	46/359 (13%)	5/63 (8%)	.40
Asian	4/359 (1%)	5/63 (8%)	**.005**
American Indian	4/359 (1%)	0/63 (0%)	1.0
Stage I	62/346 (18%)	4/59 (7%)	**.035**
Stage II	106/346 (31%)	23/59 (39%)	.23
Stage III	119/346 (34%)	18/59 (31%)	.66
Stage IV	59/346 (17%)	14/59 (23%)	.27
CIMP-0	177/251 (70%)	30/50 (60%)	.18
CIMP-low	57/251 (23%)	11/50 (22%)	1.0
CIMP-high	17/234 (7%)	9/50 (18%)	**.02**
CMS1	5/291 (2%)	2/49 (4%)	.27
CMS2	149/291 (51%)	20/49 (41%)	.22
CMS3	41/290 (14%)	7/49 (14%)	1.0
CMS4	96/290 (33%)	20/49 (41%)	.33

*P* values were calculated for comparisons between *APC*^*mut*+^ CRCs and *APC*^*mut–*^ CRCs from the TCGA dataset. A two-sample t-test with a two-tailed *p* value was performed for continuous features and a Fisher’s exact test with a two-tailed *p* value was performed for categorical data. A *p* value threshold of 0.05 was considered significant. CIMP, CpG island methylator phenotype was defined by unsupervised clustering as reported by Guinney et al*.* CMS, consensus molecular subtypes of colorectal cancer determined by Guinney et al. Significant values are in bold.

### WNT signaling mutations in ***APC***^***mut–***^ CRCs

**Figure 1:**
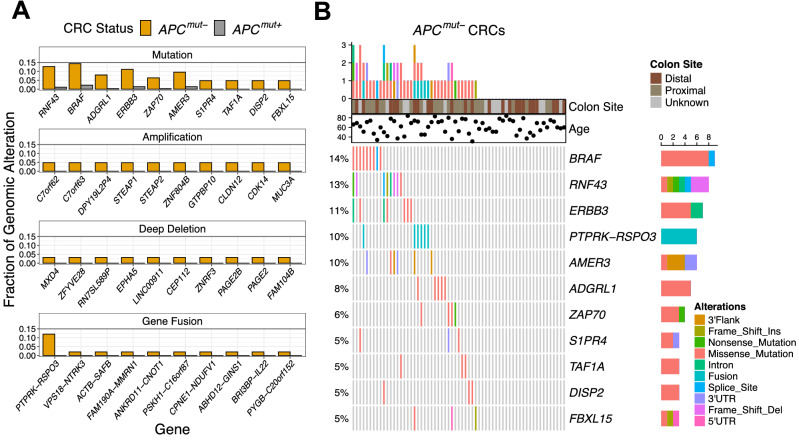
WNT signaling mutations in *APC*^*mut–*^ CRCs. (**A**) Fraction of *APC*^*mut–*^ CRCs from the TCGA dataset with gene mutations, amplifications, deep deletions, and fusions that were significantly more common in *APC*^*mut–*^ in comparison to *APC*^*mut*+^ CRCs. The top 10 are shown by p-value ranking, most significant (left) to least (right). (**B**) OncoPrint diagram showing the top 10 statistically significant mutations associated with *APC*^*mut–*^ CRCs and the gene fusion *PTPRK-RSPO3* for the 63 *APC*^*mut–*^ CRCs.

To identify distinctive somatic mutations, we compared non-silent nucleotide variants, gene amplifications, deep gene deletions, and gene fusions in *APC*^*mut–*^ and *APC*^*mut*+^ CRCs (Fig. 1A). The top three most statistically different genomic alterations specific to *APC*^*mut–*^ CRCs were *PTPRK-RSPO3* gene fusions (p = 1.3 × 10^–5^), *RNF43* mutations (p = 4.7 × 10^–5^) and *BRAF* mutations (p = 1.9 × 10^–4^). These genetic alterations have been identified in CRC previously with evidence for mutual exclusivity with *APC* mutations^27–30^. (The *RNF43* mutation G659Vfs*41, which is associated with MSI CRCs, was not present in the tumors analyzed here as MSI tumors were excluded from this analysis^31^). Eight of nine *BRAF* mutated *APC*^*mut–*^ CRCs had the oncogenic V600E *BRAF* mutation. Six of eight *RNF43* mutated *APC*^*mut–*^ CRCs had mutations that caused premature protein truncations, whilst one sample had a previously identified missense mutation, R554G. These findings suggested that *BRAF* and *RNF43* mutations are associated with tumor progression in MSS *APC*^*mut–*^ CRCs.

Based on the mutated genes in Fig. 1A and the *PTPRK-RSPO3* gene fusion, we found that only 37 out of 63 samples (59%) contained a genomic alteration that distinguished *APC*^*mut–*^ from *APC*^*mut*+^ CRCs (Fig. 1B). No pairwise combination of genes were statistically mutually exclusive. However, *PTPRK-RSPO3* gene fusions and *RNF43* mutations never co-occurred and were found in 23% of the *APC*^*mut–*^ CRCs. After disregarding overlapping genomic alterations, *BRAF* mutations were the next most abundant (10%), followed by mutations in *ADGRL1* (6%), *ERBB3* (5%), and *ZAP70* (5%). Supporting these findings, we found that *BRAF* mutations in the GSE35896 dataset and mutations in *RNF43*, *ERBB3*, and *ZAP70* in the CPTAC-2 dataset were more frequent in *APC*^*mut–*^ CRCs than in *APC*^*mut*+^ CRCs (Supplementary Fig. 1). (No additional mutation information was provided with the GSE35896 dataset.)

### Enhanced sensitivity to extracellular WNT in ***APC***^***mut–***^ CRCs

**Figure 2:**
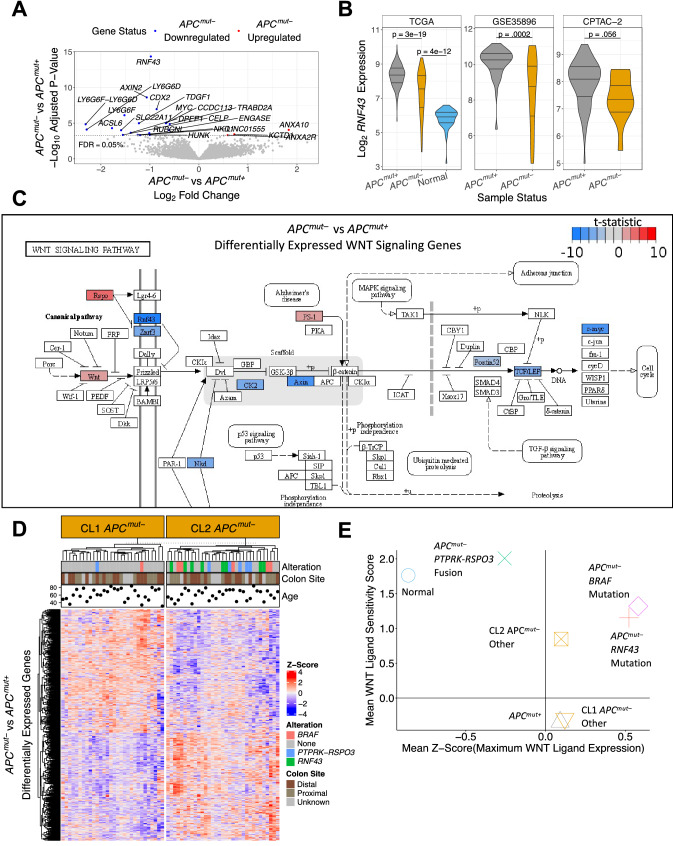
Enhanced sensitivity to extracellular WNT in *APC*^*mut–*^ CRCs. (**A**) Volcano plot representing the results from differential expression analysis between *APC*^*mut–*^ and *APC*^*mut*+^ CRCs. Labeled points are the genes with an P_adj_ < 0.0005. Blue points were downregulated in *APC*^*mut–*^ CRCs and red points upregulated. (**B**) Comparison of *RNF43* gene expression in *APC*^*mut–*^ CRCs, *APC*^*mut*+^ CRCs, and normal colon samples in the TCGA, GSE35896 and CPTAC-2 datasets. Two-sample t-tests with a two tailed p-value were used to test statistical significance. (**C**) Differentially expressed genes (P_adj_ < 0.05) between *APC*^*mut–*^ and *APC*^*mut*+^ CRCs from TCGA were mapped onto the KEGG canonical WNT signaling pathway. Blue labeling represents genes downregulated in *APC*^*mut–*^; red labeling represents upregulated genes. (**D**) Unsupervised clustering analysis of *APC*^*mut–*^ CRCs from the TCGA dataset using differentially expressed genes (P_adj_ < 0.05). (**E**) Scatter plot showing estimation of activation potential of extracellular WNT signaling. Each point is the mean for individual groups. The y-axis represents a group’s apparent sensitivity to extracellular WNT signaling using the WNT ligand sensitivity score. The x-axis represents a group’s WNT stimulation potential by quantifying each sample’s maximum WNT ligand expression.

Because a distinctive somatic mutational mechanism was not evident in over 40% of *APC*^*mut–*^ CRCs, we examined transcriptomics data for further distinguishing molecular characteristics. Strikingly, in differential gene expression analysis of the TCGA dataset, *RNF43* was the most differentially expressed gene between the two tumor groups (P_adj_ = 4.6 × 10^–15^; Fig. 2A), with a -0.98 log_2_ fold decrease in mean expression level in *APC*^*mut–*^ CRCs. Consistent with these results, *RNF43* was also down-regulated in *APC*^*mut–*^ CRCs in the GSE35896 and CPTAC-2 validation datasets (Fig. 2B). *RNF43* and its family member *ZNRF3* are membrane-bound E3 ubiquitin ligases that actuate the degradation of low-density-lipoprotein-related protein (LRP)-FZD WNT receptors. Binding of R-spondins to leucine-rich repeat-containing G-protein coupled receptors (LGR) leads to sequestration and membrane clearance of RNF43 and ZNRF3 from the cell surface^32–34^. The transcriptional down-regulation of *RNF43* we found in *APC*^*mut–*^ CRCs suggested that these tumors may express higher levels of LRP-FZD receptors at the cell surface, and consequently be more responsive to extracellular WNTs.

Because WNT signaling was implicated by these results, we sought to determine the extent to which other factors in the canonical WNT signaling pathway were differentially expressed between *APC*^*mut–*^ and *APC*^*mut*+^ CRCs (Fig. 2C). Consistent with the results above, we observed that other genes involved in extracellular WNT signaling were dysregulated, namely *RSPO3* and *ZNRF3*. Differences in *RSPO3* and *ZNRF3* mRNA expression showed a similar trend in the validation datasets and were statistically significant in select cases (Supplementary Fig. 2). We did not observe any differential expression of the extracellular WNT regulator genes *LGR4*, *LGR5*, *LGR6,* or LRP-FZD receptors. The fact that *LGR4-6* were not differentially expressed between *APC*^*mut–*^ and *APC*^*mut*+^ CRCs was consistent with the finding that *RSPO3* does not require interaction with LGRs to potentiate WNT signaling^35^ and LRP-FZD receptor levels are regulated post-transcriptionally^36^. When we compared gene expression of *APC*^*mut–*^ and *APC*^*mut*+^ CRCs to normal samples and mapped genes onto the canonical WNT signaling pathway, changes in gene expression in WNT signaling were similar between these two tumor types (Supplementary Fig. 3). These results suggested that both types of CRCs exploit changes in WNT signaling. However, based on the mutation and expression data, *APC*^*mut–*^ CRCs appear to favor dysregulation of genes involved in response to extracellular WNT signaling, whereas *APC*^*mut*+^ CRCs are stuck in the “on” state and are WNT signal-transduction incompetent.

To determine the fraction of *APC*^*mut–*^ CRCs that operate via enhanced sensitivity of extra-cellular WNT, we performed unsupervised hierarchical clustering using all differentially expressed genes (P_adj_ < 0.05) between *APC*^*mut–*^ and *APC*^*mut*+^ CRCs in the TCGA dataset (Fig. 2D). *APC*^*mut–*^ CRCs clustered into two prominent groups, referred to here as Cluster 1 (CL1) and Cluster 2 (CL2). Most *APC*^*mut–*^ CRCs with a *PTPRK-RSPO3* fusion, *BRAF* mutation, or *RNF43* mutation were in CL2. To characterize the expression profiles of *APC*^*mut–*^ CRCs in the context of extracellular WNT signaling, we computed a summarized score defined as *RSPO3* expression minus the sum of *RNF43* and *ZNRF3* expression. This score represents a theoretical WNT ligand sensitivity (WNT_LS_) based on the known function of *RSPO3* in increasing ligand sensitivity, and *RNF43* and *ZNRF3* in decreasing ligand sensitivity^33,34^. We examined how the WNT_LS_ score tracked with maximum WNT ligand expression (Fig. 2E; see Methods for more details). Consistent with our expectation, *APC*^*mut–*^ CRCs with *RNF43* mutations had higher WNT_LS_ scores than *APC*^*mut*+^ CRCs and higher maximum WNT expression than normals. Interestingly, *APC*^*mut–*^ CRCs with *PTPRK-RSPO3* fusions had the highest WNT_LS_ score, but had the lowest expression of WNT ligands compared to other CRCs. Inconsistencies in how CRCs with *PTPRK-RSPO3* fusions and CRCs with *RNF43* mutations enhance their sensitivity to extracellular WNT signaling may be due to different selective pressures during cancer evolution.

*APC*^*mut–*^ CRCs with *BRAF* mutations also exhibited higher WNT_LS_ and higher WNT ligand expression, similar to *APC*^*mut–*^ CRCs with *RNF43* mutations. Importantly, *APC*^*mut–*^ CRCs from CL2 that did not have *BRAF* mutations, *RNF43* mutations, or *PTPRK-RSPO3* fusions exhibited higher WNT_LS_ scores compared to *APC*^*mut*+^ CRCs. In contrast, *APC*^*mut–*^ CRCs from CL1 exhibited WNT_LS_ scores similar to *APC*^*mut*+^ CRCs. *APC*^*mut–*^ CRCs from the GSE35896 and CPTAC-2 datasets also clustered into two groups with high and low WNT_LS_ scores (Supplementary Fig. 4). Given the importance of WNT signaling in CRC, these results suggest that other WNT-related mechanisms drive CL1 *APC*^*mut–*^ CRCs. By transcriptomic analysis, CL1 *APC*^*mut–*^ CRCs were practically indistinguishable from *APC*^*mut*+^ CRCs; however, GSEA showed enrichment of oxidative phosphorylation genes (Supplementary Figs. 5 and 6), implicating mitochondrial activation in CL1 *APC*^*mut–*^ tumorigenesis. These results were supported by data from the DepMap CRISPR screen that demonstrated dependence of *APC*^*mut–*^ CRC cell lines on oxidative phosphorylation complexes in the mitochondria (Supplementary Fig. 5E).

### ***APC***^***mut–***^ CRCs associated with immune infiltration

**Figure 3:**
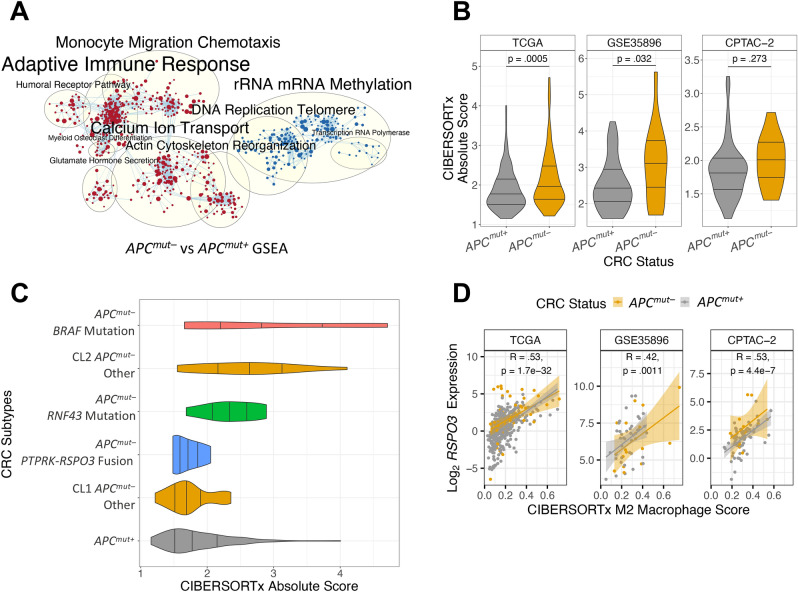
*APC*^*mut–*^ CRCs associated with immune infiltration. (**A**) GSEA results of differential gene expression analysis of *APC*^*mut–*^ versus *APC*^*mut*+^ CRCs from the TCGA dataset. Red clusters represent GO terms enriched among upregulated genes in *APC*^*mut–*^ CRCs and blue clusters correspond to down-regulated processes. (**B**) CIBERSORTx absolute score in CRCs from the TCGA, GSE35896 and CPTAC-2 datasets. Two-sample t-tests with a two-tailed p-value were used to test statistical significance. (**C**) Violin plot of CIBERSORTx absolute score across subtypes of *APC*^*mut–*^ CRCs. (**D**) Expression of *RSPO3* in *APC*^*mut–*^ and *APC*^*mut*+^ CRCs plotted against their individual M2 macrophage scores identified from the CIBERSORTx algorithm. Pearson correlation was performed to determine statistical significance.

GSEA analysis showed that GO terms related to the adaptive immune response were upregulated in *APC*^*mut–*^ compared to *APC*^*mut*+^ CRCs (Fig. 3A). To further investigate immune system involvement in *APC*^*mut–*^ CRCs, we employed the bulk tissue deconvolution method CIBERSORTx^22^. In agreement with the GSEA results, the CIBERSORTx absolute score was increased in *APC*^*mut–*^ compared to *APC*^*mut*+^ CRCs in all three CRC datasets (Fig. 3B). The CIBERSORTx absolute score was highest in *APC*^*mut–*^ CRCs with *BRAF* or *RNF43* mutations and CL2 *APC*^*mut–*^ CRCs without mutations (Fig. 3C). Because these *APC*^*mut–*^ CRCs had more infiltrating immune cells than those with *PTPRK-RSPO3* fusions, we tested whether any of the 22 immune cell types were associated with expression of WNT agonist ligand *RSPO3* (Fig. 3D). We found that M2 macrophages had the strongest positive Pearson correlation with *RSPO3* expression. M2 macrophages and *RSPO3* expression were also significantly correlated in the GSE35896 and the CPTAC-2 datasets. Macrophage expression of *RSPO3* was shown in a study of patients with pulmonary fibrosis^37^.

### ***APC***^***mut–***^ CRCs have higher ***AXIN2*** methylation

**Figure 4:**
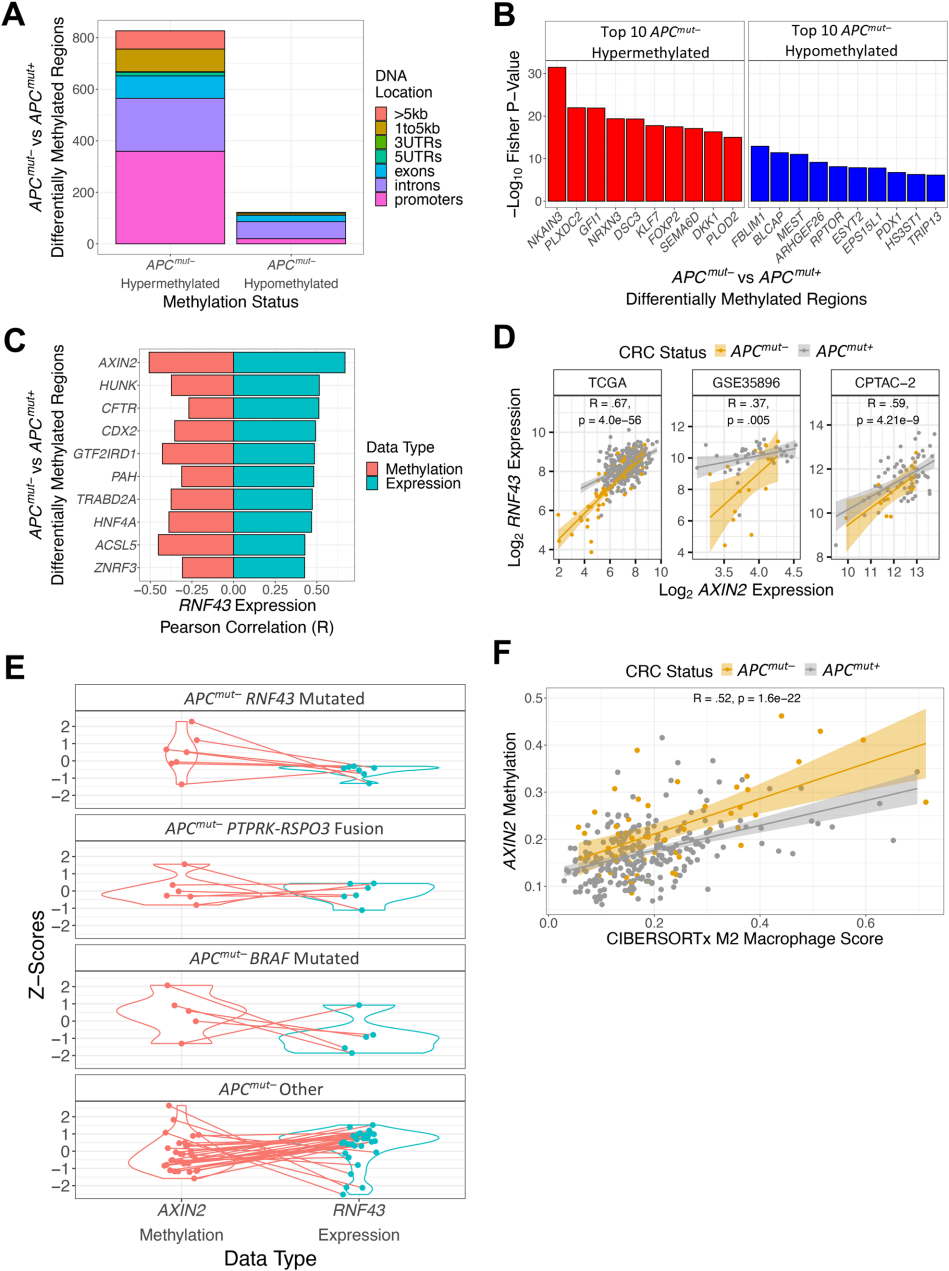
*APC*^*mut–*^ CRCs have higher *AXIN2* methylation. (**A**) Bar plot comparing total number of hyper-methylated and hypo-methylated differentially methylated regions (DMRs) between *APC*^*mut–*^ and *APC*^*mut*+^ CRCs from the TCGA dataset. (**B**) Top 10 *APC*^*mut–*^ hypermethylated and hypomethylated DMRs between *APC*^*mut–*^ and *APC*^*mut*+^ CRCs from TCGA. Red bars represent *APC*^*mut–*^ CRC differentially hypermethylated genes and blue bars represent *APC*^*mut–*^ CRC differentially hypomethylated genes. (**C**) Bar plot representing DMRs with strongest correlations with *RNF43*. Blue bars represent the top 10 DMRs with the highest Pearson gene expression correlation with *RNF43* gene expression. Red bars represent the Pearson correlation between the average differentially methylated beta values and *RNF43* expression for these differentially methylated regions. (**D**) Scatter plots of *RNF43* expression and *AXIN2* expression of both *APC*^*mut–*^ and *APC*^*mut*+^ CRCs in the TCGA, GSE35896, and CPTAC-2 datasets. Pearson correlation was performed to determine statistical significance. (**E**) Matched comparison between Z-normalized *AXIN2* average beta values and Z-normalized *RNF43* expression of *APC*^*mut–*^ CRCs. (**F**) Scatter plot of *AXIN2* average beta values and the CIBERSORTx M2 macrophage score of *APC*^*mut–*^ and *APC*^*mut*+^ CRCs from the TCGA dataset. Pearson correlation was performed to measure statistical significance.

Because we found an association between *APC*^*mut–*^ CRCs and CIMP-high previously^7^, we identified differentially methylated regions (DMRs) between *APC*^*mut–*^ and *APC*^*mut*+^ CRCs. *APC*^*mut–*^ CRCs were globally more hypermethylated than *APC*^*mut*+^ CRCs, with a particular excess in promoter regions (Fig. 4A). Comparing the top ten hypermethylated and hypomethylated DMRs, we did not observe the same statistically significant genes as we did in the mutation and expression analyses (Fig. 4B). However, when we tested correlation of *RNF43* expression with DNA methylation levels of DMRs and with RNA expression, we found that methylation and gene expression of *AXIN2* had the highest correlations (Fig. 4C). *RNF43* gene expression was also significantly correlated with *AXIN2* expression in the GSE35896 and CPTAC-2 datasets (Fig. 4D; methylation data was not available in these datasets). Increased *AXIN2* DNA methylation was associated with decreased *RNF43* expression in a subset of *APC*^*mut–*^ CRCs that did not have one of the common somatic mutations (Fig. 4E). Similar to our findings with *RSPO3* expression, we found that M2 macrophages correlated most with *AXIN2* DNA methylation (Fig. 4F).

### ***AP2M1*** gene expression associated with earlier onset in ***APC***^***mut–***^ CRCs

**Figure 5:**
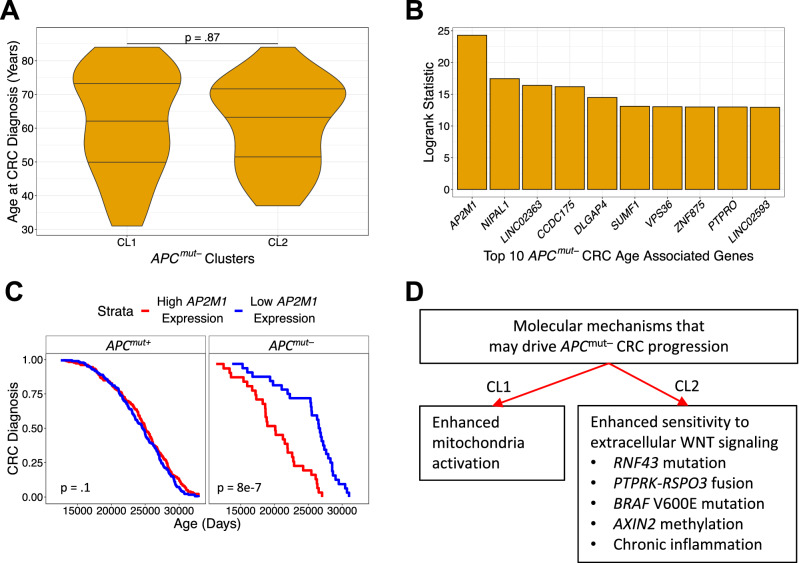
*AP2M1* gene expression associated with earlier-onset in *APC*^*mut–*^ CRCs. (**A**) A comparison of age between *APC*^*mut–*^ clusters identified from Fig. 2D. A two-sample t-test with a two-tailed p-value was used to determine statistical significance. (**B**) Top 10 statistically significant genes based on a logrank test whose median gene expression best separates age of CRC diagnosis of *APC*^*mut–*^ CRCs from the TCGA dataset. (**C**) Kaplan–Meier plot representing association between the age at CRC diagnosis and median separation of *AP2M1* expression in *APC*^*mut–*^ and *APC*^*mut*+^ CRCs from the TCGA dataset. (**D**) Flowchart of two molecular mechanisms that may be involved in the development of *APC*^*mut–*^ CRC.

Age of onset was not different in CL1 and CL2 *APC*^*mut–*^ CRCs (Fig. 5A). To identify gene expression changes linked to earlier age of onset in *APC*^*mut–*^ CRCs, we separated *APC*^*mut–*^ CRCs into two groups based on the median expression of each gene and performed a logrank test between these two groups, using the age at diagnosis as the event variable. Expression of *AP2M1* best distinguished earlier onset *APC*^*mut–*^ CRCs from later onset *APC*^*mut–*^ CRCs (Fig. 5B), and higher *AP2M1* expression was associated with earlier onset in *APC*^*mut–*^ relative to *APC*^*mut*+^ CRCs (Fig. 5C).

## Discussion

Most CRCs are initiated by somatic mutation of the gene *APC,* leading to ligand-independent, constitutive activity of the WNT pathway. In the present study, we found two alternate ways in which *APC*^*mut–*^ CRCs may activate the WNT pathway. *APC*^*mut–*^ tumors clustered into two groups according to their transcriptomic profiles (Fig. 5D). One cluster (CL2) exhibited a variety of molecular alterations that were consistent with the hypothesis that these tumors have enhanced sensitivity to extracellular WNT ligands. In particular, the most significant change was downregulation of *RNF43*, which is expected to result in increased levels of WNT receptors and greater sensitivity to extracellular WNTs. *AXIN2* methylation was highly correlated with *RNF43* downregulation. AXIN2 and RNF43 are negative regulators of WNT signaling that are transcriptionally activated by nuclear β-catenin, consistent with the notion that epigenetic silencing of negative regulators plays a critical role in tumor formation in ligand-dependent, *APC*^*mut–*^ CRCs. Similarly, *PTPRK-RSPO3* gene fusions drive R-spondin signaling, which is also expected to reduce RNF43 levels at the cell surface, upregulate WNT receptors, and enhance sensitivity to extracellular WNTs. We defined a WNT ligand sensitivity score to quantify this signature of extracellular WNT signaling in a sample-specific fashion and found a high WNT_LS_ score was associated with CL2 *APC*^*mut–*^ CRCs in multiple independent datasets.

Germline mutations in *RNF43* have been previously associated with serrated polyposis families, and somatic mutations in *RNF43* and *BRAF* have been associated with sporadic serrated adenomas^38^. In a preliminary analysis, we found that CL2 *APC*^*mut–*^ CRCs expression profiles appear to be more similar to serrated adenomas than CL1 *APC*^*mut–*^ CRCs and *APC*^*mut*+^ CRCs, according to two published gene signatures^39,40^ (data not shown), but these results need further investigation.

We also found that CL2 *APC*^*mut–*^ CRCs have a higher level of immune infiltration compared to *APC*^*mut*+^ and CL1 *APC*^*mut–*^ CRCs, especially in *APC*^*mut–*^ CRCs that had *RNF43* or *BRAF* mutations. M2 macrophages had the strongest association with potentiating WNT signaling through its significant correlations with *RSPO3* expression and *AXIN2* DNA methylation. Previous studies have shown that macrophages have the capability to express *RSPO3* and stimulate WNT signaling in response to tissue damage^41,42^. The association of CL2 *APC*^*mut–*^ CRCs with M2 macrophages suggests the etiology of this cancer subtype is tied to chronic tissue stress and inflammation that eventually favors a clone with hypersensitivity to WNT. We suggest that CL2 *APC*^*mut–*^ CRCs may be sensitive to porcupine inhibitors or anti-WNT/anti-DKK1 biologics. We note that *AXIN2* methylation has been previously identified in *APC*^*mut–*^ CRCs as a potential biomarker for ligand-dependent tumors that would respond to anti-WNT-based therapies such as porcupine inhibitors^43–45^.

In contrast, the other cluster (CL1) of *APC*^*mut–*^ CRCs was associated with low WNT_LS_ score and may be dependent on enhanced mitochondrial activation. *APC*^*mut–*^ CRC cell lines from the DepMap database had a strong dependency on mitochondrial activation relative to *APC*^*mut*+^ CRC cell lines. We are cautious in interpreting these data, because the observed effectiveness of mitochondrial disruption of the *APC*^*mut–*^ CRC cell lines may be due to the absence of immune cells in vitro. One potential reason why some *APC*^*mut–*^ CRCs become dependent on enhanced mitochondrial activation is because mitochondria can stimulate the WNT pathway independently of WNT ligands^46^. Moreover, intestinal epithelial cell-specific knockout of TFAM, a transcription factor required for replication of mitochondria DNA, drastically reduced tumor formation in *APC*^*min/*+^ mouse models^47^. Therefore, we suggest that enhanced activation of mitochondria is a second, independent mechanism by which *APC*^*mut–*^ CRCs exploit WNT signaling in tumor progression. These findings also suggest that mitochondria inhibitors may be a promising therapeutic option for CL1 *APC*^*mut–*^ CRCs.

Although *APC*^*mut-*^ tumors overall exhibit a lower age of onset than APC^*mut*+^ tumors, we found no difference in age of onset between CL1 and CL2, suggesting that both extracellular WNT sensitivity and mitochondrial activation contribute to the incidence of early-onset CRC. We performed a *APC*^*mut–*^wide analysis to determine what gene expression feature was most associated with age of onset and found that earlier-onset *APC*^*mut–*^ CRCs had increased expression of *AP2M1*. *AP2M1* plays an important role in clathrin-mediated endocytosis^48^. A recent study showed that when insulin binds to an insulin receptor, *IRS1* and *IRS2* recruit *AP2M1* to initiate insulin receptor endocytosis^49^. Thus, an increase of *AP2M1* may suggest increased insulin signaling. Importantly, insulin can activate both the PI3K pathway and the MAPK pathway, which may in turn play a role in enhancing both mitochondrial activation and immune infiltration, thus contributing to driving both CL1 and CL2 subtypes of *APC*^*mut–*^ CRC^50–52^. Other studies have found that individuals with type two diabetes are at a greater risk for early-onset CRC^53,54^.

Early-onset CRC is a rapidly advancing public health emergency, and it is associated with a lack of mutation in *APC*. Our comprehensive genomic analysis has uncovered two classes of *APC*^*mut–*^ CRCs, one which potentiates WNT signaling through sensitivity to extracellular signaling, and the other which exhibits mitochondrial activation. Future research should test the effect of anti-WNT biologics and mitochondrial inhibitors in organoid models and in vivo and compare the efficacy of *AXIN2* methylation and WNT ligand sensitivity score in identifying anti-WNT sensitive tumors.

## Supplementary Information

Supplementary Information 1.

Supplementary Information 2.

Supplementary Information 3.

Supplementary Figures.

## Data Availability

Data analyzed in this study can be found in the Genomic Data Commons (https://gdc.cancer.gov); Gene Expression Omnibus (GSE35896; https://www.ncbi.nlm.nih.gov/geo/); cBioPortal (CPTAC-2; https://www.cbioportal.org/); and DepMap (https://depmap.org/portal/). All analytic methods and study materials are available to other researchers through supplemental materials and in the methods section.
