# Supplementary material for: Molecular drivers of tumor progression in microsatellite stable APC mutation-negative colorectal cancers
Source: Sci Rep. 2021 Dec 6;11:23507. doi: 10.1038/s41598-021-02806-x (PMC8648784; doi:10.1038/s41598-021-02806-x)
Supplement: Supplementary file 4 — Supplementary Figures. [file 41598_2021_2806_MOESM4_ESM.pdf]

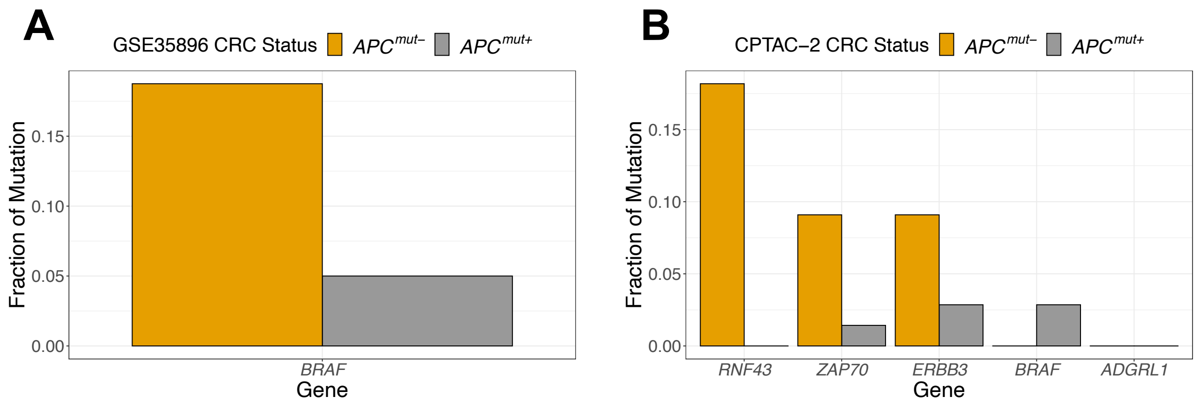

Supplementary Figure 1. Mutation occurrence in validation datasets. (*A*) The fraction of *BRAF* mutations in *APC*$^{mut-}$ and *APC*$^{mut+}$ CRCs from the GSE35896 dataset. (*B*) The fraction of *RNF43*, *ZAP70*, *ERBB3*, *BRAF* and *ADGRL1* mutations in *APC*$^{mut-}$ and *APC*$^{mut+}$ CRCs from the CPTAC-2 dataset.

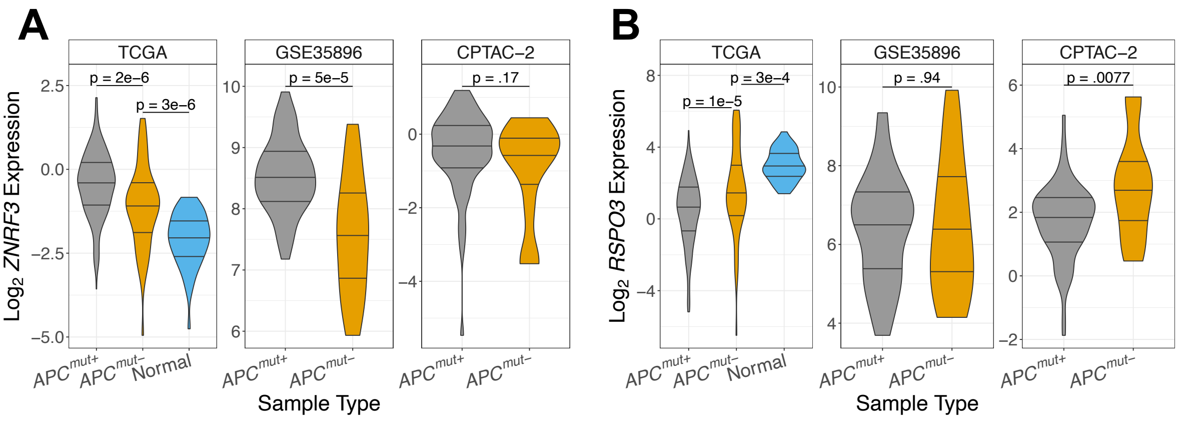

Supplementary Figure 2. Comparison of ZNRF3 and RSPO3 expression in discovery and validation datasets. Comparison of *ZNRF3* expression (*A*) and *RSPO3* expression (*B*) between APC^{mut-} CRCs, APC^{mut+} CRCs, and normal colon samples from the TCGA, GSE35896, and CPTAC-2 datasets. A two-sample t-test with a two-tailed p-value was used to determine statistical significance.

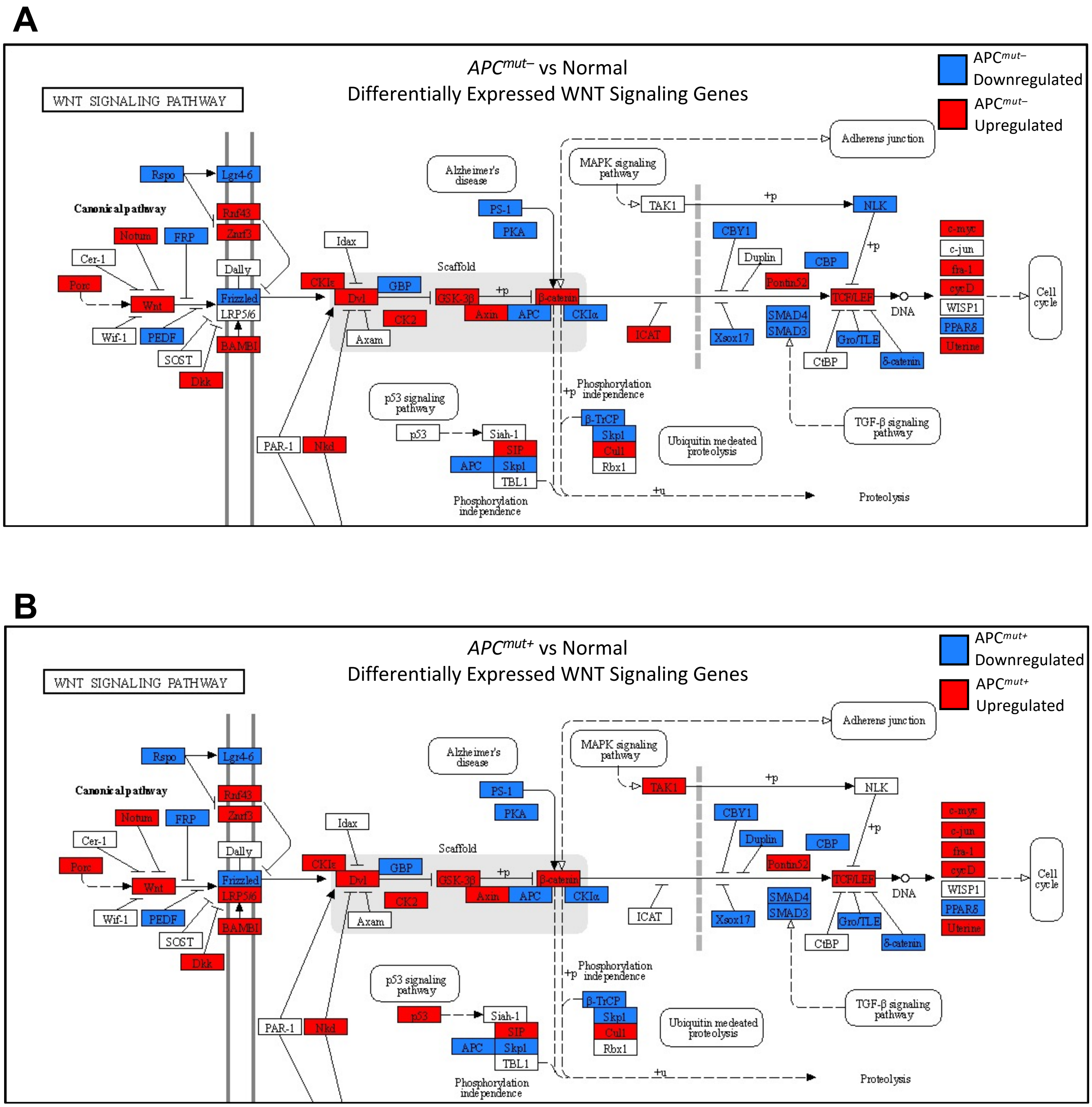

Supplementary Figure 3. WNT pathway alterations in tumors relative to normal colon tissue. Differentially expressed genes ($P_{adj} < 0.05$) between *APC*$^{mut-}$ CRCs and normal colon samples (*A*) and *APC*$^{mut+}$ CRCs and normal samples (*B*) were mapped onto the KEGG canonical WNT signaling pathway. Blue labeled genes represent downregulation relative to normal samples, while red labeled genes represent upregulation relative to normal colon samples.

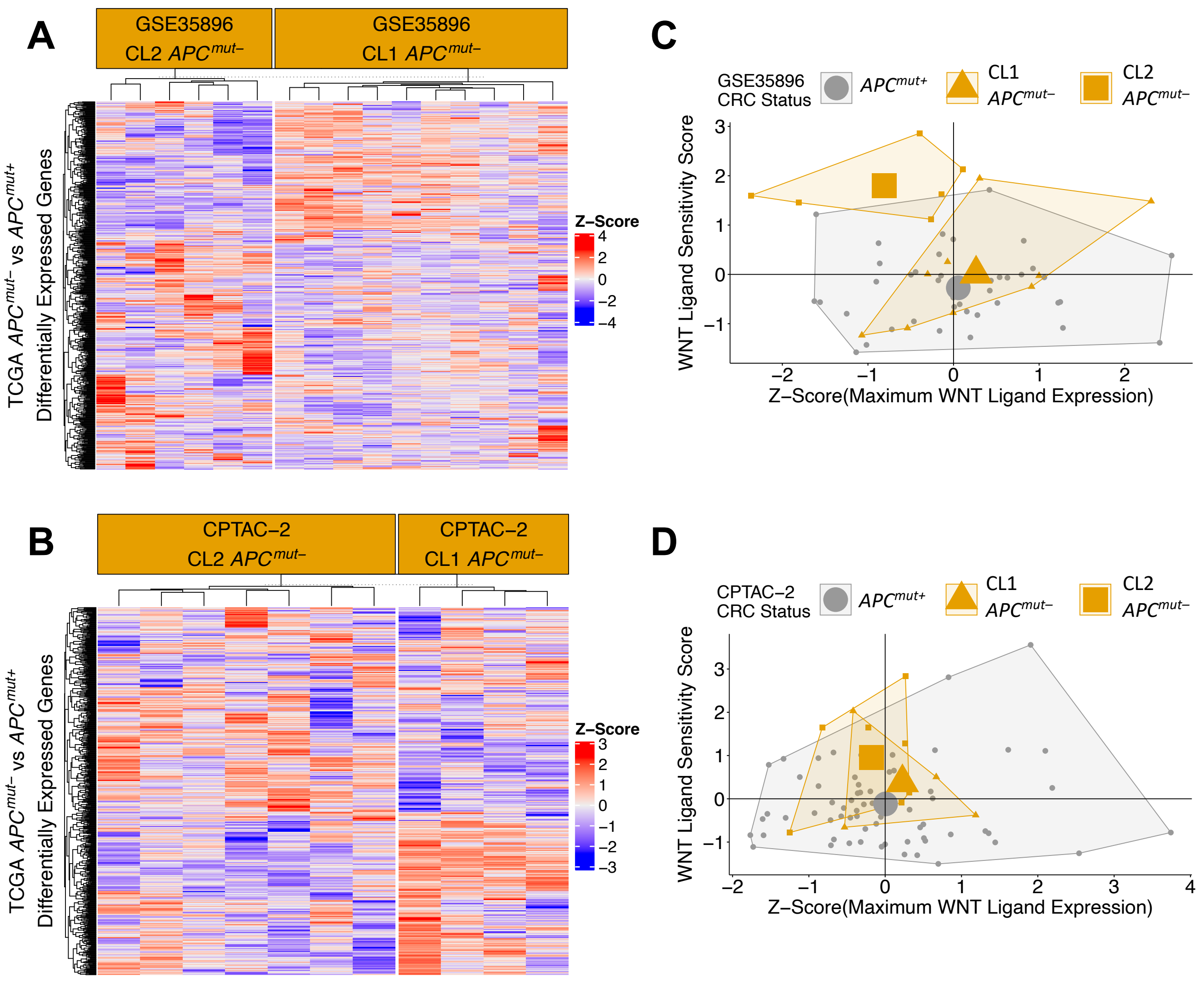

Supplementary Figure 4. Extracellular WNT sensitivity in validation datasets. Unsupervised clustering of APC^{mut-} CRCs from the GSE35896 dataset (*A*) and the CPTAC-2 dataset (*B*) using differentially expressed genes identified from comparing APC^{mut-} and APC^{mut+} CRCs from TCGA. Scatter plots for the GSE35896 dataset (*D*) and the CPTAC-2 dataset (*E*) showing the estimation of activation potential of extracellular WNT signaling for APC^{mut-} CL1, APC^{mut-} CL2, and APC^{mut+} CRCs. The y-axis represents a sample's apparent sensitivity to extracellular WNT signaling using the WNT ligand sensitivity score. The x-axis represents a sample's WNT stimulation potential by quantifying each sample's maximum WNT ligand expression.

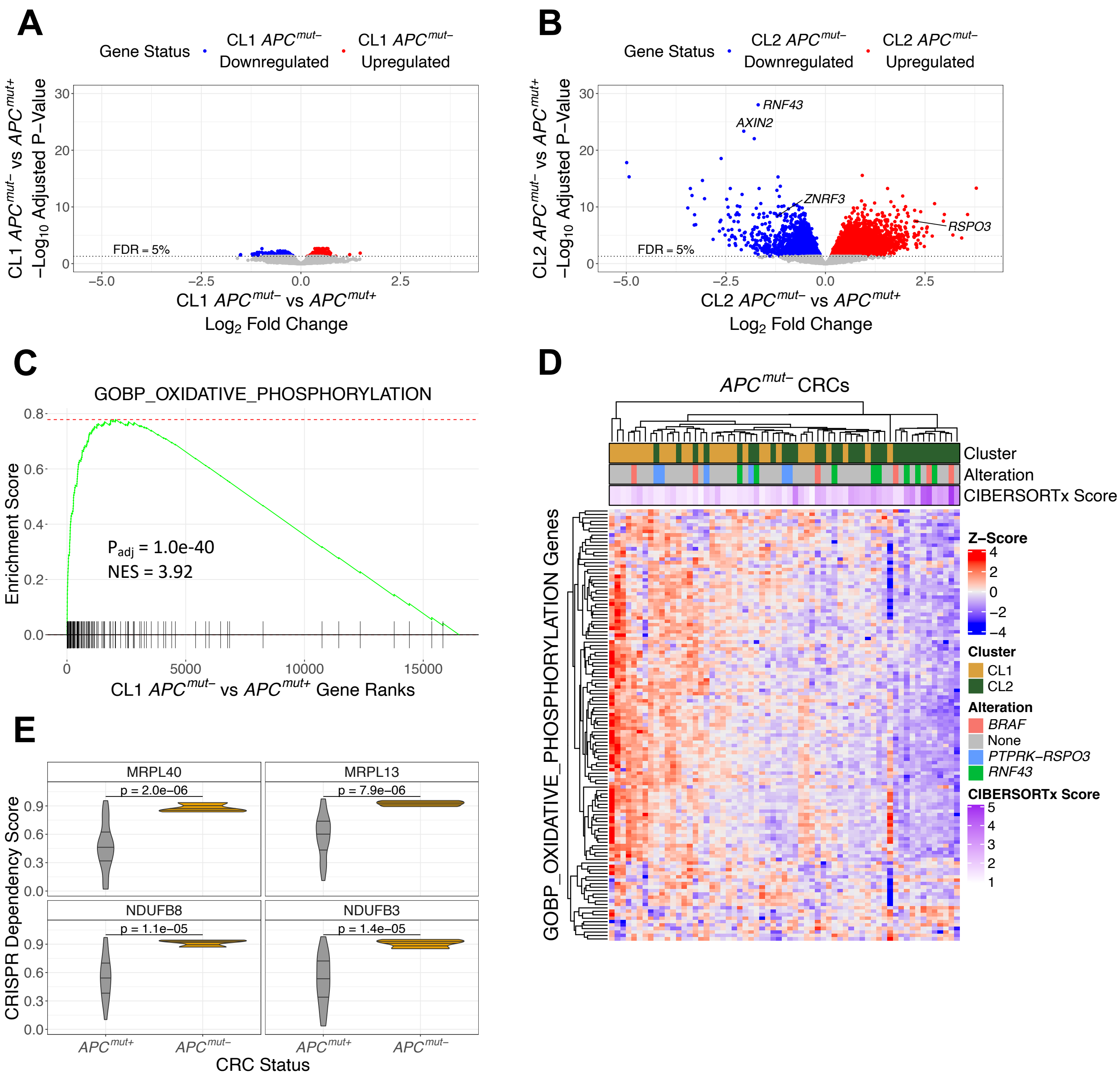

Supplementary Figure 5. Enhanced mitochondrial activation in CL1 APC^{mut-} CRCs. *(A)* A volcano plot representing differential expression analysis between CL1 APC^{mut-} and APC^{mut+} CRCs from the TCGA dataset. Blue points were downregulated in CL1 APC^{mut-} CRCs and red points upregulated. (*B*) A volcano plot representing differential expression analysis between CL2 APC^{mut-} and APC^{mut+} CRCs from the TCGA dataset. Blue points were downregulated in CL2 APC^{mut-} CRCs and red points upregulated. *(C)* An enrichment plot of the most significant upregulated GO term from GSEA analysis between CL1 APC^{mut-} and APC^{mut+} CRCs from the TCGA dataset. (*D*) Unsupervised cluster analysis of APC^{mut-} CRCs from the TCGA dataset using genes associated with the biological process GO term Oxidative Phosphorylation. Shown are each APC^{mut-} CRC's CIBERSORTx absolute score (representing total number of estimated infiltrating immune cells), presence of a *PTPRK-RSPO3* fusion, *BRAF* or *RNF43* mutation and CL1 or CL2 cluster from Figure 2D. (*E*) Violin plots of CRISPR dependency scores of the mitochondria-related genes *MRPL40*, *MRPL13*, *NDUFB8*, *NUDFB3* in APC^{mut-} CRC cancer cell lines (n = 3) and APC^{mut+} CRC cancer cell lines (n = 16). A Welch's two-sample t-test with a "greater than" alternative hypothesis was used to test for statistical significance.

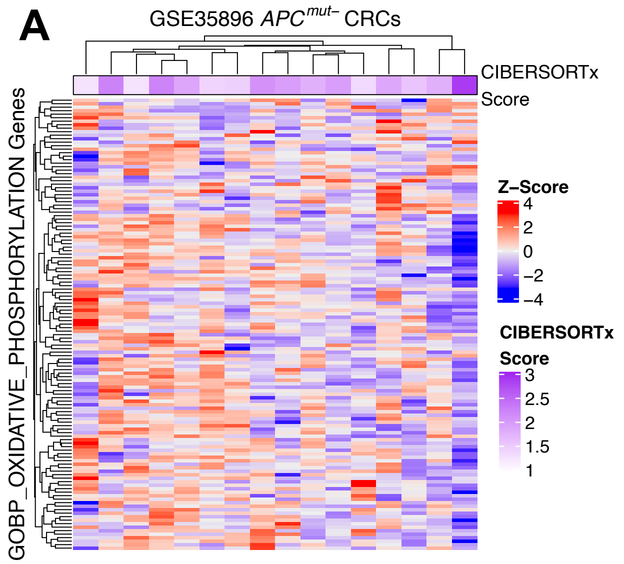

Supplementary Figure 6. Oxidative phosphorylation and immune infiltration in validation datasets. Unsupervised cluster analysis of *APC*$^{mut-}$ CRCs from the GSE35896 dataset *(A)* and the CPTAC-2 dataset *(B)* using genes associated with the biological process GO term Oxidative Phosphorylation. Shown are each *APC*$^{mut-}$ CRC's CIBERSORTx absolute score representing total number of estimated infiltrating immune cells.
